# Colorectal adenocarcinoma with hepatic neuroendocrine carcinoma: A case report

**DOI:** 10.1097/MD.0000000000035428

**Published:** 2023-11-03

**Authors:** Lingzi Shi, Li Sun

**Affiliations:** a Department of Gastrointestinal Surgery, Xi’an Jiaotong University School of Medicine Affiliated Honghui Hospital, Xi’an, China.

**Keywords:** case report, clinical differential diagnosis, hepatic neuroendocrine carcinoma, Liver metastasis of colorectal adenocarcinoma

## Abstract

**Rationale:**

Primary hepatic neuroendocrine tumors are rarely reported and extremely blurry to diagnose, especially in the case of a confirmed diagnosis of colon cancer and a family history. Here we report such a case followed by our experiences and lessons.

**Patient concerns:**

A 62-year-old male with a family history of colon cancer has been recently admitted to our hospital, exhibiting multiple hepatic lesions when diagnosed as colon cancer, and all assistant examinations indicated the hepatic metastases.

**Diagnoses:**

Liver puncture biopsy and immunohistochemistry confirmed hepatic neuroendocrine carcinoma, which was tended to primary hepatic tumor combining medical history.

**Interventions and outcomes:**

The patient refused the further treatment and dead of liver failure.

**Lessons:**

Hepatic neuroendocrine tumors exhibited no specific symptoms, signs or imaging manifestations, mainly relying on immunohistochemistry for diagnosis, which makes it difficult to be distinguished from other liver masses and metastatic tumors, especially interfered by a confirmed diagnosis of colon cancer and a family history. In this regard, more rigorousness is required in the diagnosis and treatment of liver tumors.

## 1. Introduction

Neuroendocrine neoplasms (NENs) refer to a primary malignant tumor arising from neuroendocrine cells, which can be found throughout the body due to it widespread distribution.^[1]^NENs occurring in the liver are classified as hepatic neuroendocrine neoplasms (HNENs), mostly referring to metastatic hepatic neuroendocrine neoplasms, for which the primary focus still remains the gastrointestinal lesion itself.^[2]^ Primary hepatic neuroendocrine neoplasms (PHNENs) are rarely reported, with a limited number of cases in all types of literature since the first description by Edmondson in 1958, accounting for only 0.3% to 4.0% of all NENs and approximately 0.28% to 0.46% of liver malignancies 0.28% to 0.46% of all liver malignancies.^[3]^In the present case, an adenocarcinoma of the colon combined with small cell neuroendocrine carcinoma of the liver was reported, followed by the review of related literatures.

## 2. Case presentation

**Figure 1.**
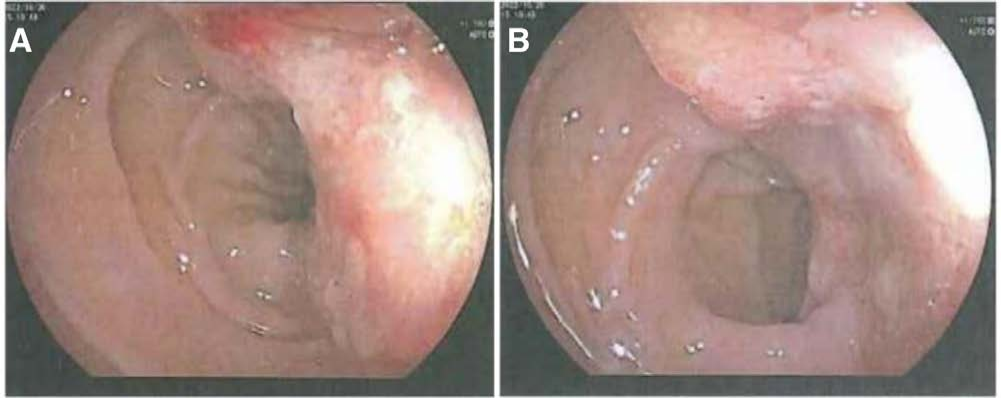
(A and B) Enteroscopy showed irregular mucosal bulge accounting for 1/2 of the canal lumen was seen at the sigmoid colon junction 40 cm from the anus, with friable tissue and easy bleeding when taken biopsy.

**Figure 2.**
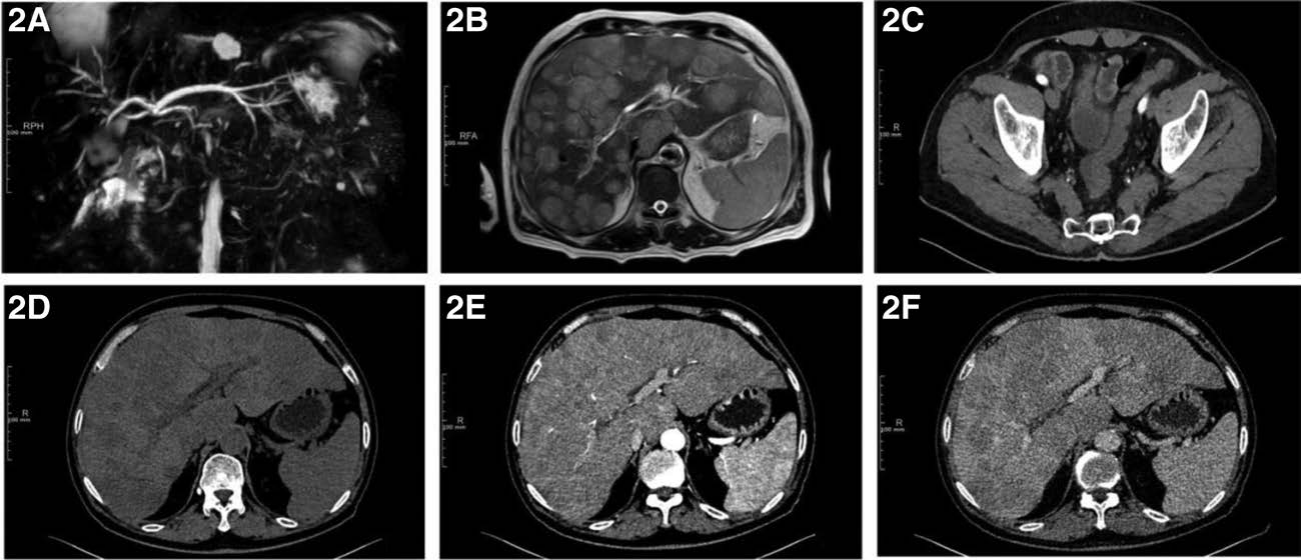
(A) MRCP showed that the common hepatic duct and the upper part of the common bile duct are not clearly visible, and the end of the common bile duct is faintly visible. (B) MRCP also showed the diffuse heterogeneous lamellar slightly long-long T2 signal shadow in the liver. (C) Computerized tomography (CT) showed sigmoid colon is stiff with narrow lumen and lymph node shadow around it. (D–F) There were low and slightly low density on plain scan CT and progressive enhancement when enhanced in Liver. CT = computerized tomography, MRCP = magnetic resonance cholangiopancreatography.

**Figure 3.**
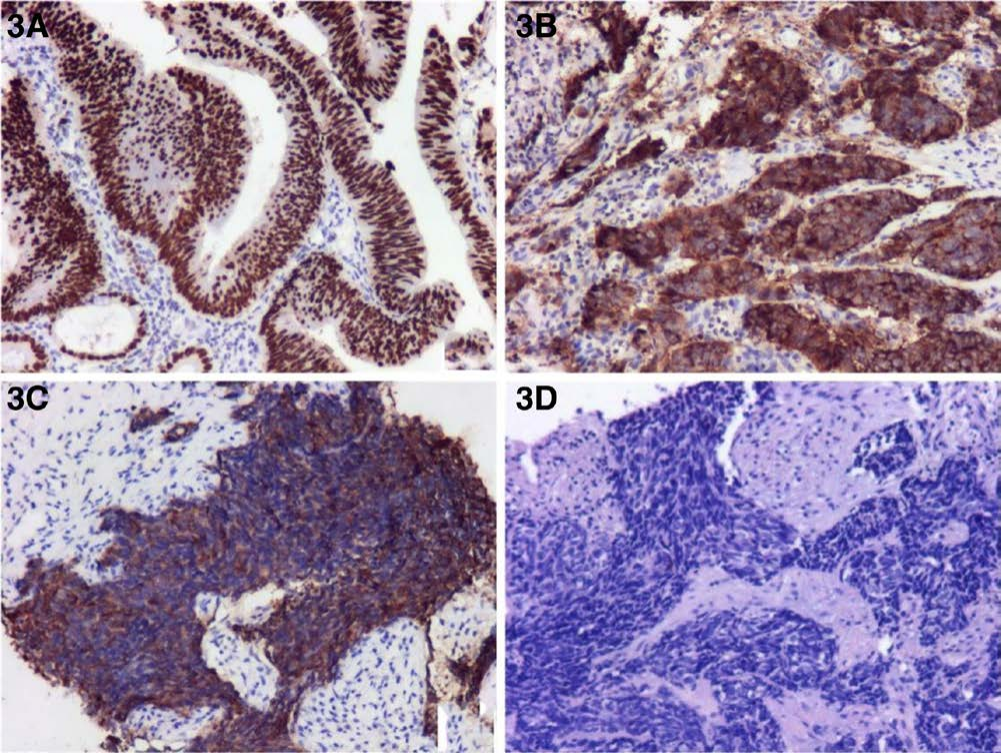
(A and B) Immunohistochemical staining of sigmoid colon mass biopsy (×100) positive nuclear staining can be seen, which suggested CDX-2(+). (C and D) Liver puncture pathology and immunohistochemistry (×100): Syn (+), NSE (+), Ki-67 (about 70%), showing small cell type neuroendocrine carcinoma.

The patient is a 62-year-old male, presented with diarrhea for 2 months and yellowing of the skin all over the body for 1 month. He reported the diarrhea averaging 3 to 6 times/day with mucus or pus-blood stools, without improvement after symptomatic treatment. The weight has dropped by 8.5 kg during 2 months, without pruritus in circumferential skin, the NRS2002 score revealed 3 and an ECOG score of 0. Physical examination: general condition was fair, general skin and sclera were yellowish, no skin lesions, no palpable enlargement of general lymph nodes, no abnormalities in cardiopulmonary examination, no positive signs in abdominal examination or anal finger diagnosis. No special past was reported. Family history: The elder and younger brother had been diagnosed as colon cancer. On admission, hematological indices: Glutathione transaminase 61.2 U/L, glutathione transaminase 96.0 U/L, glutamyl transferase 1129.0 U/L, alkaline phosphatase 544.4 U/L, total bilirubin 111.0 mmol/L, direct bilirubin 101.1 mmol/L, indirect bilirubin 9.9 mmol/L, total bile acid 98.73 mmol/L, cholinesterase 3487U/L; AFP 4.97 ng/mL, CEA 1.50 ng/mL, CA125 149.6 U/mL, CA199 32.03 U/mL, CA72-4 13.87 U/mL, NSE 279.1 ng/mL, non-small cell lung cancer related antibody 19.35 ng/mL (Table 1). Enteroscopy: irregular mucosal elevation with rough and uneven surface was seen at the sigmoid junction 40 cm from the anus, occupying 1/2 of the canal lumen, without resistance to endoscopic passage, the material was brittle and easily bleeding when touched (Fig. 1). Magnetic Resonance Cholangiopancreatography: no exact visualization of the common hepatic duct and the upper end of the common bile duct was observed, with the faintly visualized end of the common bile duct; diffuse heterogeneous lamellar slightly long-long T2 signal shadow in the liver (Fig. 2A and B). Enhanced computerized tomography (CT) of the chest, whole abdomen and pelvis: the sigmoid colon was stiff, the intestinal wall was thickened with the narrowed lumen, the mucosa was not continuous, the intestinal wall was moderately enhanced, with the enhanced surrounding lymph node shadow; the liver exhibited multiple hypointense shadows, with progressive enhancement (Fig. 2C–F). Liver ultrasonography suggested hypoperfusion type. Enteroscopic biopsy pathology and immunohistochemistry: (descending colon – sigmoid junction) medium-hypodifferentiated adenocarcinoma; CK (+), CDX-2 (+), CK20 (+), P53 (mutant +), CK7 (partial cell +), CgA (-), Syn (focal +), HER-2 (1+), EGFR (+), MLH1 (+), MSH2 (+), MSH6 (+), PMS2 (+), Ki67 (about 70%) (Fig. 3A and B). Liver puncture pathology and immunohistochemistry: small cell type neuroendocrine carcinoma; AE1/AE3 (+), Hepatocyte (-), AFP (-), CK20 (-), CK19 (+), CK7 (-), CgA (scattered +), Syn (+), NSE (+), Ki-67 (about 70%) (Fig. 3C and D). The present obstructive jaundice due to diffuse intrahepatic tumor of the patient was an absolute contraindication to adjuvant therapy such as surgery and chemotherapy, and the MDT discussion tended to no obvious effect by the present effective yellowing reduction tools (including PTCD, ERCP, and ultrasound endoscopy-guided biliary drainage).

**Table 1:** Laboratory data on admission.

Blood count		Blood chemistry		Tumor markers	
WBC (3.5–5.5)	8.15 × 10^9^/L	ALT (9–50)	92.3 U/L	AFP (0–8.1)	4.97 ng/mL
RBC (4.3–5.8)	3.58 × 10^12^/L	AST (15–40)	125.0 U/L	CEA (0–5)	1.50 ng/mL
NEUT# (2–7.7)	6.08 × 10^9^/L	S/L	1.4	CA125 (0–35)	149.6 U/mL
NEUT% (45–77)	74.7%	GGT (10–60)	1164.0 U/L	CA153 (0–25)	10.81 U/mL
LYMPH# (0.8–4.0)	1.0 × 10^9^/L	ALP (45–125)	882.5 U/L	CA199 (0–27)	32.03 U/mL
LYMPH% (20–40)	12.3%	TBIL (1.7–17.1)	249.0 µmol/L	CA72–4 (0–6.9)	13.87 U/mL
EO# (0.05–0.5)	0.03 × 10^9^/L	DBIL (0–6.9)	221.4 µmol/L	TPSA (0–4)	0.929 ng/mL
EO% (0.5–5.0)	0.3%	IBIL (1.7–10.2)	27.6 µmol/L	FPSA (0–0.93)	0.3 ng/mL
BASO# (0.0–0.1)	0.02 × 10^9^/L	TP (65–85)	67.5 g/L	F/P	0.32
BASO% (0.0–1.0)	0.2%	ALB (40–55)	36.7 g/L	FERRITIN (30–400)	493.20 ng/mL
HGB (130–175)	108g/L	GLOB (20–40)	30.8 g/L	CYFRA21–1 (0–3.3)	19.35 ng/mL
HCT (40–50)	32.60	A/G (1.2–2.4)	1.2 Ratio	NSE (0–16.3)	279.1 ng/mL
PLT (125–350)	236 × 10^9^/L	TBA (0–10)	318.07 µmol/L	ProGRP (≤65.7)	40.2 pg/mL
		CHE (5000–12,000)	3196 U/L		

AFP = alpha-fetoprotein, ALB = albumin, ALP = alkaline phosphatase, ALT = alanine aminotransferase, AST = aspartate transaminase, BASO = basophilicgranulocyte, CA = carbohydrate antigen, CEA = carcinoembryonic antigen, CHE = cholinesterase, CYFRA21-1 = cytokeratin 19 fragment antigen21-1, DBIL = direct bilirubin, EO = eosnophils, FPSA = free prostate specific antigen, GGT = γ-glutamyl transpeptadase, GLOB = globulin, HCT = red blood cell specific volume, HGB = hemoglobin, IBIL = Indirect bilirubin, LYMPH = lymphocyte, NEUT = neutrophil, NSE = neuron specific enolase, PLT = platelet, ProGRP = pro-gastrin-releasing peptide, RBC = red blood cell, TBA = total biliary acid, TBIL = total bilirubin, TP = total protein, TPSA = total prostate specific antigen, WBC = white blood cell.

## 3. Discussion

### 3.1. Primary hepatic neuroendocrine tumors

NENs as the primary malignant tumors arising from neuroendocrine cells are most commonly reported in the gastrointestinal tract, followed by the respiratory system and the thymus.^[4]^ NENs could be classified into G1, G2 and G3 stages according to nuclear schizophrenia, and G3 stage with Ki67 over 20% is called neuroendocrine carcinoma,^[5]^about 0.28% to 0.46% of liver malignancies.^[4]^ A certain body of scholars suggest that PHNENs cells are derived from ectopic pancreas in the liver, adrenal tissue or dispersed neuroendocrine cells in the epithelium of the intrahepatic bile duct;^[6]^and another hypothesis considering neuroendocrine-oriented differentiation of a single malignant stem cell as the main cause of PHNENs generation.^[6]^

PHNENs are mainly manifested by occupational symptoms such as pain in the liver area, right upper abdominal pain, poor nausea, vomiting, and abdominal masses; metastatic HNENs with diverse clinical manifestations could be divided into functional and nonfunctional types, with the former mostly presenting with clinical symptoms related to tumor secretion of 5-hydroxytryptamine metabolites and peptide hormones,^[7]^ such as refractory ulcers, Whipple syndrome, Cushing’s syndrome, and neurogenic hypoglycemia; the latter with atypical gastrointestinal symptoms such as abdominal distension, nausea and lethargy, vomiting of blood and black stools, and jaundice.^[2]^ However, most patients are admitted not for cancer-like symptoms,^[8]^ possibly due to the low incidence of hormone-related manifestations resulting from insufficient production or defective secretion of hormones and their metabolites. The presence of jaundice implies not only the massive hepatocellular invasion but also the tumor compression of the intra- and extra-hepatic bile ducts, which generally results from the coexistence of disease features obstructive and hepatocellular jaundice.

HNENs usually exhibit no characteristic tissue lesion, where the ancillary tests other than pathology has tentative contribution as diagnosis. While the direct choice of biopsy in a suspected malignancy also increases the risk of tumor spread, posing a critical requirement of clinicians’ experience plus the extremely low incidence of HNENs. HNENs have a diverse imaging presentation, easily presenting large, isolated or massive growths with lobulated or irregular contours that appear as small non-enhanced lesions on CT and high intensity lesions on T2-weighted images on magnetic resonance imaging,^[9]^ and hyper-echoic masses similar to hemangiomas in ultrasound, with a “fast-in, fast-out” pattern on 80% of ultrasound imaging.^[10]^ PHNENs are frequently revealed by single or lobulated giant lesions, or multiple foci confined to the right lobe of the liver, which may be related to the flow pattern shunting of blood from the superior mesenteric vein into the liver. PHNENs in early stage usually present with intrahepatic metastases, while with rare distant metastases; metastatic HNENs tumors are categorized by swollen growth masses in the right and left lobes of the liver without differences. In terms of laboratory tests, the variation in liver function and tumor markers of PHNENs are usually insignificant, while metastatic HNENs generally exhibited the elevated CEA,CA199 and NSE as well as the declined liver function.^[2]^No significant differences in pathological manifestations and immunohistochemistry are exhibited between both of them and are mainly differentiated by medical history and other imaging examinations.^[11]^ The diagnosis of PHNENs mainly relies on pathology with extrahepatic primary foci strictly excluded, PHNENs are pathologically difficult to be differentiated from metastatic hepatic neuroendocrine neoplasms due to the homology, while requiring comprehensive examination and long-term follow-up to exclude any potential extrahepatic primary lesions for diagnosis.

Surgery remains the primary treatment, just as lobectomy or liver transplantation; local ablation, TACE, and cytotoxic drugs^[12]^ serve as options for non-surgical treatment, and the efficacy of mTOR inhibitors and small-molecule TKI drugs or biological agents such as growth inhibitor analogs requires more research.

### 3.2. Liver metastasis from colon cancer

Approximately 60% to 70% of colon cancer patients are revealed to exhibited liver metastases at the time of first visit to a doctor, becoming the leading cause of death in this disease. In addition to the nonspecific gastrointestinal manifestations appearing in the middle and late stages, hematological tests such as CA19-9, CEA, human heat shock protein 90α in combination with ALP, GGT, LDH may serve as a reference for screening liver metastases in patients with colon cancer.^[13]^ In terms of imaging, enhanced CT mainly reveals nodular or ring-like enhancement with uniform distribution of liver size, but also single or giant mass-like, with charactered by manifestations such as bull’s eye and wheel signs; ultrasonography reveals oligovascular metastatic liver cancer and multivessel metastatic liver cancer, which matches the characteristics of blood supply to the primary focus, most commonly in adenocarcinoma with predominantly venous blood supply. The diagnosis can be basically confirmed by clinical manifestations, hematological and imaging examinations combined with the history of primary colon cancer. But atypical manifestations in some patients may lead to misdiagnosis, so liver aspiration biopsy is still required in an obscure judgment. Jaundice in liver metastatic cancer indicates the end stage of tumor, with the causes and types similar to those mentioned above.

The resectable metastatic colorectal cancer relies on surgery, while inoperable patients may opt for local treatment (including radiofrequency ablation, microwave ablation, or stereotactic radiotherapy) as well as pharmacotherapy. Studies have demonstrated a high similarity of cancer cell characteristics at metastatic sites to those of the primary site, while clinical treatment and medication are not yet recommended to exactly refer to the primary site considering the rapid metabolism of hepatocytes and tumor heterogeneity,^[14]^ and more evidence-based medical evidence is required.

### 3.3. Identification and reflection

Combining the pathology of the colon mass and the family history of colon cancer in this paper, it was initially more inclined to consider the liver lesion as metastatic cancer rather than the primary. Meanwhile, the liver function tests demonstrated the more obvious elevation of GGT and AKP compared to AST or ALT, accompanied by obstructive jaundice alterations, also suggesting the potential metastatic liver lesion; the normality of AFP and CEA in the tumor markers could also exclude the liver A normal AFP and CEA in tumor markers excludes primary cancer, while an abnormal elevation of NSE may guide a consideration of the origin of the mass. When considering the puncture results, the hepatic occupancy was primary rather than metastatic.

Up to now, the treatment of liver metastases from colon cancer has been relatively standardized, but more cautious is still required in clinical diagnosis. In the context of the patient’s treatment, that the liver lesion is a metastasis from colon cancer is rather unlikely, but it still cannot be completely excluded considering the family history, medical history and tumor heterogeneity; the immunohistochemistry of the patient’s gastrointestinal lesion did not suggest neuroendocrine component, which highly indicates the intrahepatic metastasis of liver lesion in PHNENs rather than in gastrointestinal NENs, while the process of disease development still requires exploration. Therefore, the choice of subsequent treatment is controversial. It is a rare case, and no systematic treatment plan for this disease has been found according to literatures. Combining the adjuvant examination and MDT discussion, surgery was unavailable, and symptomatic treatment was adopted for the time being, rescue chemotherapy could be considered after the general condition had improved, and the plan was more inclined to etoposide + cisplatin and other cytotoxic chemotherapy drugs or small molecule monoclonal antibodies.^[15]^

In conclusion, NENs-related liver lesions, without characteristic symptoms, are rarely reported especially when accompanied by the primary disease and easy to misdiagnose, when the patient does not have a history of hepatitis or cirrhosis, the negative AFP and elevated NSE should be combined with imaging and immunohistochemistry before making a judgment, empirical diagnosis should be abandoned to avoid a wrong judgment.

## Author contributions

**Writing – original draft:** Lingzi Shi.

**Writing – review & editing:** Li Sun.
